# The Secret Life of Translation Initiation in Prostate Cancer

**DOI:** 10.3389/fgene.2019.00014

**Published:** 2019-01-30

**Authors:** Greco Hernández, Jorge L. Ramírez, Abraham Pedroza-Torres, Luis A. Herrera, Miguel A. Jiménez-Ríos

**Affiliations:** ^1^Translation and Cancer Laboratory, Unit of Biomedical Research on Cancer, National Institute of Cancer, Mexico City, Mexico; ^2^Cátedra-CONACyT Program, Hereditary Cancer Clinic, National Institute of Cancer, Mexico City, Mexico; ^3^Unidad de Investigación Biomédica en Cáncer, Instituto Nacional de Cancerología-Instituto de Investigaciones Biomédicas, The National Autonomous University of Mexico, Mexico City, Mexico; ^4^Department of Oncologic Urology, National Institute of Cancer, Mexico City, Mexico

**Keywords:** prostate cancer, translation initiation, translational control, androgen receptor, eIF4E, eIF4G, mTOR, MAPK

## Abstract

Prostate cancer (PCa) is the second most prevalent cancer in men worldwide. Despite the advances understanding the molecular processes driving the onset and progression of this disease, as well as the continued implementation of screening programs, PCa still remains a significant cause of morbidity and mortality, in particular in low-income countries. It is only recently that defects of the translation process, i.e., the synthesis of proteins by the ribosome using a messenger (m)RNA as a template, have begun to gain attention as an important cause of cancer development in different human tissues, including prostate. In particular, the initiation step of translation has been established to play a key role in tumorigenesis. In this review, we discuss the state-of-the-art of three key aspects of protein synthesis in PCa, namely, misexpression of translation initiation factors, dysregulation of the major signaling cascades regulating translation, and the therapeutic strategies based on pharmacological compounds targeting translation as a novel alternative to those based on hormones controlling the androgen receptor pathway.

## Introduction

Among different types of cancers, prostate cancer (PCa) is the third most commonly diagnosed tumor around the world, ranking second in incidence among men and fifth leading cause of cancer death in this gender. The most recent data (2018) have reported about 360,000 deaths and almost 1.3 million new cases due to this neoplasia worldwide (Dy et al., 2017; Bray et al., 2018; Ferlay et al., 2018; Pilleron et al., 2018). In low-income countries, the importance of this malady is even more dramatic. For instance, in the Americas, PCa is the most commonly diagnosed malign neoplasia with over 400,000 new cases and the second cause of cancer death with about 80,000 dead men in 2018 (Global Burden of Disease Cancer Collaboration et al., 2015; Dy et al., 2017; Bray et al., 2018; Pilleron et al., 2018).

Prostate is a gland laying underneath the bladder that secretes factors for sperm maintenance and viability throughout life. PCa is defined as the uncontrolled growth of cells from the gland epithelium that acquire the ability to scatter. Indeed, PCa is a highly heterogeneous disease, comprising mostly adenocarcinomas that display a wide spectrum of both clinical evolution patterns and phenotypic defects (Humphrey, 2014; Seitzer et al., 2014; Network, 2015; Packer and Maitland, 2016; Arora and Barbieri, 2018). Nowadays, the parameters most used for surveillance, diagnosis, and design of treatments are the blood level of prostate-specific antigen (PSA), the biopsy clinical stage, and the Gleason score of tumors (Humphrey, 2014; Seitzer et al., 2014; Jones et al., 2018; Martin et al., 2018; McCrea et al., 2018).

Prostate cancer pathology has started to be understood at the molecular level. Normal development and function of prostate strongly depend on the action of both, androgens and androgen receptor (AR, a transcriptional factor). Most tumors exhibit AR gene amplification and/or somatic prostate mutations (Gottlieb et al., 2012). Thus, AR malfunctioning may be a main trigger for the onset and progression of PCa. Other genes are also found dysregulated in PCa. For example, loss of one copy of the tumor suppressor *PTEN* has been reported in nearly 60% of PCa patients (Phin et al., 2013), which appears to be a critical component in the evolution of PCa with metastasic potential (Baca et al., 2013). In metastasic prostate tumors, amplification of the oncogene *c-myc* (DeVita et al., 2011) and mutations in the genes involved in cell cycle regulation *Cyclin-dependent kinase inhibitor 1B* (*CDKN1B*) and *TP53* (Baca et al., 2013) have also been reported. Moreover, promoter hypermethylation of different genes such as *PTEN, retinoblastome gene* (*RB*), and *cadherin 1 gene* (*CDH1*) has been linked to advanced stages of PCa (Friedlander et al., 2012).

Genomic rearrangements involving the 5′ untranslated region (UTR) of *E26 transformation-specific* (*ETS*) gene family members also occur in approximately 50% of PCa tumors (Rubin et al., 2011). A DNA rearrangement found in 40–50% of primary PCa tumors produces TMPRSS2-ERG, the fusion of the *androgen induced transmembrane gene serine 2 protease* gene (*TMPRSS2*) with members of the *erythroblast transformation-specific related gene* (*ERG*) family of transcription factors (Tomlins et al., 2005), which results in the androgen-dependent *ERG* oncogenic expression (Nam et al., 2007; Perner et al., 2007; Tu et al., 2007; Albadine et al., 2009; Fine et al., 2010).

Advanced PCa tumors are regularly treated by hormone-deprivation via different types of castration to block AR function. However, this eventually leads to treatment resistance and the tumor recurs as a castration-resistant prostate cancer (CRPC). Unfortunately, studies on the CRPC condition are scarce. Some AR splicing variants lacking regulatory regions, such as the ligand-binding domain, contribute to the development of CRPC (Guo et al., 2009; Hu et al., 2009). Comparisons between primary PCa and CRPC revealed significant differences in *ERG* expression, with primary tumors displaying higher expression levels (Roudier et al., 2016). This may indicate that *ERG* expression is important in primary PCa and may no longer be required in CRPC tumors that might use a different mechanism to promote proliferation and cell survival (Roudier et al., 2016). Moreover, genome sequencing of CRPC tumors have shown that the most recurrently alterations are mutations in the *TP53* and *AR* genes, the *TMPRSS2:ERG* fusion, loss of *RB* and *breast cancer gene* (*BRCA*) genes, and gains in *AR* and *MYC* copy numbers (Grasso et al., 2012). In contrast, these genomic alterations were less frequent among clinically localized primary tumors, supporting the idea that hormonal deprivation may induce changes that alter *AR* function (Taplin et al., 1999).

Translation has recently begun to gain attention as a possible key molecular process in cancer development, because cancer cells display rapid growth and proliferation with significantly increased protein synthesis. Translation is largely controlled at the initiation step and translation initiation was frequently found to be involved in the development of different types of cancer, including PCa (Parsyan, 2014; Bhat et al., 2015; Sharma et al., 2016; Truitt and Ruggero, 2016; Ali et al., 2017; Robichaud et al., 2018). Thus, targeting translation initiation is being probed as part of the global schemas of some emerging cancer therapies. Here, we review a rapidly growing field of the study of translation initiation contribution to PCa, as well as the signaling pathways regulating it. We also summarize the most relevant research on pharmacological compounds targeting translation initiation as a new potential mean to alleviate this malady.

## An Overview of Translation Initiation and Its Regulatory Signaling Cascades

Translation is a sophisticated and tightly controlled process that plays a central role in gene expression. It consists of three main stages, namely, initiation, elongation, termination, and a final stage in which the ribosome recycles. Overall, the initiation step consists of the recruitment of the 40S ribosome subunit to the 5′-UTR of an mRNA through the action of around a dozen initiation factors (eIFs) (Jackson et al., 2010; Hinnebusch, 2014; Hershey et al., 2018). This process is mostly regulated by two signaling cascades, the mTOR and the mitogen-activated protein kinase (MAPK) pathways.

### Translation Initiation

Translation initiation begins when a free 40S ribosomal subunit interacts with eIF1, eIF1A, eIF3, eIF5, and the so-called ternary complex (consisting of eIF2 bound to GTP and an initiator Met-tRNA_i_^Met^) to form a 43S pre-initiation complex (PIC). This step positions the initiator Met-tRNA_i_^Met^ in the peptidyl (P) decoding site of the ribosome. In a parallel set of reactions, the cap structure (m^7^GpppN, where N is any nucleotide) located at the 5′ end of the mRNA is recognized by eIF4E. Then, the scaffold protein eIF4G performs simultaneous interactions with the cap-bound eIF4E, the RNA-helicase eIF4A, poly(A)-binding protein (PABP), and the ribosome-bound eIF3, to coordinate recruitment of the 43S PIC to the mRNA 5′-UTR. Because PABP binds the poly(A) tail at the mRNA 3′ end, this set of interactions circularizes the translating mRNA. Then, the 43S PIC scans base-by-base the mRNA 5′-UTR to reach the AUG start codon, a process in which eIF4A, assisted by eIF4B, unwinds 5′-UTR secondary structures. Selection of the correct AUG start codon is driven by eIF1 and eIF1A, that leads to the establishment a perfect Watson–Crick match between the anticodon of the Met-tRNA_i_^Met^ and the mRNA start codon. Selection of the authentic start codon establishes the open reading frame for mRNA decoding, arrests mRNA scanning, and results in formation of a 48S PIC containing the Met-tRNA_i_^Met^ and eIF1A tightly positioned within the A-site. Afterward, GTP hydrolysis of GTP—eIF5B promotes the release of eIF5B from the 80S monoribosome, which facilitates 60S ribosomal subunit joining and the assembly of an 80S initiation complex, which is ready to start elongation (Jackson et al., 2010; Hinnebusch, 2014; Hershey et al., 2018).

### mTOR Pathway

**FIGURE 1:**
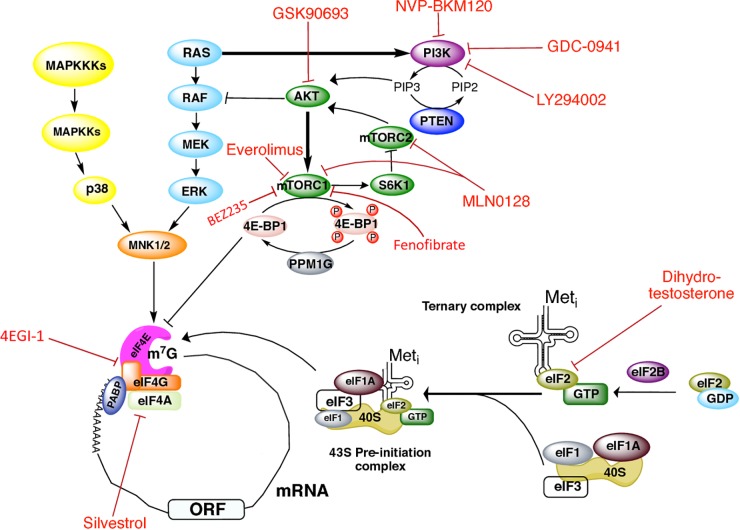
Signaling cascades regulating initiation of translation in prostate cancer and therapeutics targets. Regulation of translation initiation factors by the PI3K/AKT/mTOR, RAS-ERK, and MAPK signaling pathways, as well as the drugs that have been used in PCa to target these molecules.

Two major signaling cascades control protein synthesis, namely, the phosphatidylinositol 3-kinase (PI3K)/protein kinase B (Akt)/mammalian target of rapamycin complex 1 (mTORC1) pathway, and the mitogen-activated protein kinase (MAPK) pathway (Figure 1). The serine/threonine kinase mTOR is the core of two structurally and functionally distinct multisubunit complexes, namely, mTORC1 and mTOR2. mTORC1 is composed by the proteins lethal SEC13 protein 8 (mLST8), pleckstrin [DEP]-domain-containing mTOR interacting protein (DEPTOR), regulatory associated protein of mTOR (RAPTOR), and proline-rich Akt substrate 40 kDa (PRAS40). The mTORC1 signaling pathway senses, integrates, and responds to nutrient availability, stress, cellular energy status, hormones, and mitogens to control cellular growth, survival, and proliferation, as well as translation, transcription of ribosomal RNAs and transfer RNAs, ribosome biogenesis, lysosome biogenesis, lipid synthesis, and protein breakdown. TORC2 regulates co-translational protein degradation and cytoskeletal organization (Fonseca et al., 2016; Proud, 2018; Roux and Topisirovic, 2018). Thus, only mTORC1 is of our interest here, as mTORC1 pathway integrates cellular signals to control translation through the phosphorylation of proteins with functions in the initiation and elongation steps.

mTORC1 phosphorylates factors that directly regulate the translational machinery, as well as protein kinases that phosphorylate translation factors, including the eIF4E-binding proteins (4E-BPs) and the S6 kinases (S6Ks). mTORC1 also promotes the indirect phosphorylation of initiation factors eIF4B, eIF4G, and elongation factor 2 kinase (eEF2K) (Fonseca et al., 2016; Proud, 2018; Roux and Topisirovic, 2018). Binding of 4E-BPs to eIF4E precludes its association with eIF4G and represses cap-dependent translation. Binding to eIF4E is controlled by the phosphorylation status of 4E-BPs: whereas hypophosphorylated 4E-BPs bind eIF4E with high affinity, the hyperphosphorylated species dissociate from eIF4E to relieve translational repression. The reverse reaction is favored by the protein phosphatase 1G (PP1G) that removes 4E-BP1 phosphate groups. S6Ks control translation by modulating the activity of targets such as ribosomal protein S6, eIF4B, and programmed cell death 4 protein (PDCD4), a negative eIF4A regulator (Fonseca et al., 2016; Proud, 2018; Roux and Topisirovic, 2018).

### MAPK Pathway

The MAPKs pathway also regulates translation (Figure 1). MAPKs are serine/threonine kinases that mediate intracellular signaling associated with a variety of cellular activities, including cell proliferation, differentiation, survival, death, and transformation. MAPK cascades components are activated by mitogens and stress stimuli, and are coupled to the translation machinery via the phosphorylation of downstream MAPK-activated protein kinases (collectively known as MKs). In response to diverse stimuli (Scheper et al., 2001; Wang et al., 2001), ERK or p38 MAPK phosphorylate Mnk 1/2 kinases, which in turn interact with the carboxy-terminal of eIF4G to directly phosphorylate eIF4E on Ser-209, resulting in stimulation of translation (Proud, 2018; Roux and Topisirovic, 2018). The RAS-ERK pathway crosstalks with the PI3K/AKT/mTORC1 pathway. When bound to GTP, RAS can directly bind and allosterically activate PI3K (Mendoza et al., 2011). AKT negatively regulates ERK activation by phosphorylating RAF in its amino-terminus (Mendoza et al., 2011). ERK in turn phosphorylates RAPTOR which activates TORC1 in an AKT-independent way (Herbert et al., 2002; Foster et al., 2010; Carriere et al., 2011).

In the following, we will focus on how malfunction of eIFs and the mTOR and MAPK pathways impact PCa, and review the numerous molecular defects related to translation that have been reported in PCa (Table 1). We will also discuss the prospects of targeting translation in PCa treatments using drugs inhibiting translation.

**Table 1:** Defects in eIFs and the signaling pathways regulating translation in prostate cancer.

Protein	Defect	Reference
PI3K or MAPK cascades signaling components	(1) Point mutations and genomic alterations in *PIK3CA/B*, causing overactivation of the PI3K/Akt/mTORC1 pathway	Sun et al., 2009; Barbieri et al., 2012; Robinson et al., 2015
	(2) 25% of the prostate cancers show a presumed actionable lesion in members of the PI3K or MAPK signaling pathways	Network, 2015
	(3) Rare gene fusions found in RAF1, that could drive MAPKs pathway activation in PCa	Palanisamy et al., 2010; Wang et al., 2011; Beltran et al., 2012
PTEN	(4) Deletions and high rate of mutations in the *PTEN locus* are present in nearly 40–60% of primary PCa tumors	Barbieri et al., 2012; Beltran et al., 2012; Robinson et al., 2015
TORC2	(5) Activates AKT in PCa cells	Sarbassov et al., 2006
eIF3	(6) eIF3e is downregulated in PCa.	Marchetti et al., 2001
	(7) eIF3d expression is upregulated in PCa.	Gao et al., 2015
	(8) Overexpression of eIF3h in PCa.	Saramaki et al., 2001
eIF4E	(9) eIF4E is overexpressed and hyperphosphorylated in PCa (10) eIF4E S209 phosphorylation promotes resistance to bicalutamide treatment	Graff et al., 2009; Furic et al., 2010 D’Abronzo et al., 2017
eIF4G	(11) eIF4G1 chromosomal location (3q27.1) is amplified in PCa patients	Luo et al., 2002
4E-BP1	(12) Hyperphosphorylation correlates with poor prognosis in PCa diagnostics.	Graff et al., 2009
	(13) Critical regulator of both PCa initiation and maintenance downstream of mTOR signaling in a genetic mouse model; increased 4E-BP1 abundance observed in PCa patients	Hsieh et al., 2015


## Translation Initiation Factors Involved in PCa

### eIF2

eIF2 is composed of three subunits (α, β, and γ) that form the core of the ternary complex GTP/eIF2/Met-tRNA_i_^Met^, which delivers initiator methionyl-tRNA_i_ to the ribosomal P-site during translation initiation. eIF2α regulates protein synthesis depending on its phosphorylation status. Phosphorylated eIF2α increases its affinity for its guanine nucleotide exchange factor eIF2B, leading to the formation of inactive eIF2B–eIF2–GDP complexes that suppress cap-dependent translation. eIF2α can be phosphorylated by four stress-responsive kinases upon various stimuli, namely, double-stranded RNA activated protein kinase (PKR), general control non-repressed 2 (GCN2) kinase, heme-regulated inhibitor (HRI), and PKR like endoplasmic reticulum kinase (PERK), that become activated in response to viral infection, decreased nutrients, oxidizing agents, high salt levels, hypoxia, and heat-shock among others (Wek, 2018).

Nguyen et al. (2018) used murine and humanized models to demonstrate that PCa can respond adaptively via eIF2α phosphorylation to reset global protein synthesis and promote aggressive tumor development. Additionally, high expression of phosphorylated eIF2α along with loss of PTEN in 424 PCa patients was found to associate with increased risk of metastasis (Nguyen et al., 2018). The critical role of eIF2α phosphorylation to regulate the global rate of translation renders eIF2α a promising target for PCa treatments.

### eIF3

The multisubunit eIF3 is the largest of initiation factors, with an approximate size of 804 kDa. This factor is a complex of 13 subunits, namely, eIF3a-m, that bridges between the 43S PIC and the mRNA/eIF4F complex during translation initiation. The functions of the different eIF3 subunits are varied. While some fulfill essential tasks for the synthesis of proteins, others have regulatory activities (Hinnebusch, 2006). Of particular interest for PCa, eIF3h was frequently found overexpressed in tumors and high levels of eIF3h positively correlated with increased Gleason scores (Nupponen et al., 1999; Saramaki et al., 2001). However, overexpression of eIF3 subunits is not a rule in PCa; in fact, the eIF3e subunit was found to be down regulated in this neoplasia (Marchetti et al., 2001).

### eIF4F

The eIF4E cap-binding protein, together with the eIF4A RNA helicase and the eIF4G scaffold protein, form the eIF4F complex that drives mRNA recruitment to the 40S ribosome subunit to initiate mRNA translation. Although eIF4E is required for cap-dependent translation of all nuclear-transcribed mRNAs, some mRNAs with long and highly structured 5′-UTR have showed a high requirement for eIF4E and the eIF4A helicase unwinding activity (Rajasekhar et al., 2003; Mamane et al., 2007; Feoktistova et al., 2013). Tightly related to cancer development, these so-called “eIF4E-sensitive” transcripts encode proteins that stimulate cell survival and proliferation, such as vascular endothelial growth factor-A (VEGF-A), hypoxia inducible factor 1 alpha (HIF1-α), BCL-2 family members, ornithine decarboxylase 1 (ODC1), cyclin D3, and c-MYC (Feoktistova et al., 2013; Truitt et al., 2015; Hinnebusch et al., 2016; Truitt and Ruggero, 2016; Vaklavas et al., 2017). Their regulation is critical for normal cell proliferation. Accordingly, using a haploin-sufficient eIF4E mouse model (eIF4E+/-), Truitt et al. (2015) observed that a 50% reduction of eIF4E levels protected the animals from cellular transformation and tumorigenicity.

eIF4E, eIF4G, and eIF4B have been implicated in PCa development. Overexpression of eIF4E has been reported in advanced tumor stages, and also to be associated with decreased rates of patient survival (Wang et al., 2005; Graff et al., 2009). eIF4E phosphorylation promotes tumor development in prostate and has been found to be elevated in PCa. Moreover, eIF4E is highly phosphorylated in hormone-refractory PCa, which correlates with poor clinical outcome (Furic et al., 2010). By using a model knock-in mice expressing a non-phosphorylatable version of eIF4E, Furic et al. (2010) also demonstrated that eIF4E phosphorylation is required for translational upregulation of several mRNAs, and that increased phospho-eIF4E levels correlate with disease progression in patients with PCa.

Jaiswal et al. (2018) have observed that eIF4G1 protein levels are increased in PCa tumors as compared to normal tissues, and that gene expression of this protein positively correlates with the tumor grade and stage. Accordingly, eIF4G1 silencing impaired cell viability, proliferation, and migration and downregulated genes involved in the epithelial-mesenchymal transition, such as N-cadherin and Snail-1 (Jaiswal et al., 2018). Renner et al. (2007) have observed that eIF4G phosphorylation was increased in the prostate of transgenic mice expressing a constitutively active p110-alpha catalytic subunit of PI3K. Consistently, inhibition of PI3K activity with the drug LY294002 inhibited eIF4G phosphorylation. Thus, eIF4G phosphorylation has been proposed as a new marker for PI3K activity in PCa (Renner et al., 2007). Finally, both meta-analysis and immunoblot of tissue extracts showed that eIF4G is overexpressed in human PCa epithelial tissue (Wang et al., 2005).

eIF4B activity has been also proven to affect PCa development (Oh et al., 2007). Accordingly, the protein level of the serine/threonine kinase Proviral integration site of murine (Pim-2) was found to significantly correlate with eIF4B phosphorylation both in PCa samples and in cell lines (Ren et al., 2013). Pim-2 is a potent anti-apoptotic factor and its upregulation is associated with prostatic carcinoma tumorigenesis, suggesting that Pim-2 overexpression may cause direct eIF4B phosphorylation during PCa tumorigenesis (Ren et al., 2013).

## Dysregulation of the Major Signaling Cascades Controlling the Translation Machinery in PCa

### PI3K/Akt/mTOR Pathway

In response to different stimuli, PI3K phosphorylates phosphatidylinositol-4,5-biphosphate (PIP2) yielding phosphatidylinositol-3,4,5-triphosphate (PIP3). This reaction is balanced by PTEN, which catalyzes the reverse reaction. PIP3 acts as a second messenger propagating intracellular signals and resulting in AKT activation. Upon activation, AKT phosphorylates several proteins, including the mTORC1.

The PI3K/Akt/mTOR signaling pathway is frequently hyperactivated in most human cancers, and inactivation of tumor suppressors such as PTEN, LKB1, and TSC1/2, which antagonize the PI3K/AKT/mTORC1 pathway, may drive tumorigenesis (Fonseca et al., 2016; Proud, 2018; Roux and Topisirovic, 2018). Activation of the PI3K pathway is associated to resistance to androgen deprivation therapy and to poor outcomes in PCa (Jiao et al., 2007; Reid et al., 2010; Bitting and Armstrong, 2013; Liu and Dong, 2014). Aberrations in PI3K/AKT/mTORC1 signaling have been identified in approximately 40% of early PCa cases and 70–100% in advanced cases and metastasic tumors (Taylor et al., 2010; Carver et al., 2011). In particular, overactivation of this pathway via *PTEN* loss significantly favors initiation of PCa (Di Cristofano et al., 1998; Suzuki et al., 1998; Podsypanina et al., 1999), and leads to constitutive activation of the PI3K pathway in 60% of CRPCs (Vivanco and Sawyers, 2002). Pre-clinical data indicated that some PTEN-deficient neoplasms, including PCa, activated the PI3K pathway via the p110beta isoform of the PI3K catalytic subunit (Jia et al., 2008; Wee et al., 2008; Ni et al., 2012). Moreover, mutations of AKT or its gene amplification have also been observed in different PCa cases (Sadeghi and Gerber, 2012). Genetic studies in mouse models have implicated mTOR hyperactivation in triggering PCa *in vivo* (Guertin et al., 2009; Nardella et al., 2009). It has also been shown that 4E-BP1 may regulate tumor initiation and progression through mTOR signaling in PCa (Hsieh et al., 2015).

### MAPK/ERK Pathway

MAPK signaling is divided into three subtypes, namely, extracellular signal-regulated protein kinase (ERK), p38 MAPK, and c-Jun *N*-terminal kinase/stress-activate protein kinase (JNK/SAPK), that play a key role in modulating intracellular responses, including translation. Whereas JNK/SAPK and p38 have been generally linked to cell death and tumor suppression, ERK plays a prominent role in cell survival and tumor promotion in response to a broad range of stimuli (Zhuang et al., 2005; Proud, 2018; Roux and Topisirovic, 2018).

In most cancer types, including PCa, the MAPK signaling cascades are found hyperactivated and also play a role in tumor growth, castration-resistant development, and metastasis (Wagner and Nebreda, 2009; Mulholland et al., 2012; Rodriguez-Berriguete et al., 2012; Proud, 2018; Roux and Topisirovic, 2018). Their inhibition prevents PCa cell growth (Gioeli et al., 1999; Kinkade et al., 2008), and in *Pten-null;Ras* activated PCa cells, the RAS/MAPK pathway was observed to play a significant role in metastasis (Mulholland et al., 2012).

## Targeting Translation Initiation in PCa

A summary of the translation initiation factors as well as the components of the PI3K/AKT/mTOR pathway used as therapeutic targets in PCa is depicted in Figure 1 (Kinkade et al., 2008; Edlind and Hsieh, 2014). PI3K is a common therapeutic target with existing drugs such as GDC-0941 and LY294002 which are reported to inhibit proliferation in human (Raynaud et al., 2009) and mouse transgenic (Renner et al., 2007) PCa cell lines. NVP-BKM120, a pan-class I PI3K inhibitor, showed antiproliferative activity in xenograft animal models and the PCa cell line PC3 (Maira et al., 2012). The use of the AKT inhibitor GSK690693 has also demonstrated antitumoral activity in PCa xenograft animal models (Rhodes et al., 2008).

mTOR is perhaps the most targeted molecule in PCa. mTOR inhibition by the drug INK128 was described to prevent PCa cells invasion and metastasis *in vivo* (Guertin et al., 2009; Nardella et al., 2009). In combination with the AR inhibitor bicalutamide (not depicted), Everolimus (also termed RAD001) inhibits mTORC1 and leads to growth arrest in some castration-resistant PCa models (D’Abronzo et al., 2017). The drug MLN0128 (also known as INK128) has been reported to make a dual inhibition of both mTORC1 and mTORC2 complexes in PCa cells, preventing metastasis and inducing apoptosis (Sarbassov et al., 2006; Hsieh et al., 2012; Bitting and Armstrong, 2013). However, a recent study suggested that the clinical efficacy of MLN0128 is limited (Graham et al., 2018). Fenofibrate is a widely used drug for its lipid-lowering activity, and some reports have described its inhibitory effect on growth of different PCa cell lines, such as LN and PC-3. It induces apoptosis mediated by oxidative stress (LN cells) (Zhao et al., 2013), or by the caspase-3 and the apoptosis-inducing factor (AIF) signaling pathways (PC-3 cells) (Lian et al., 2017). In PC-3 cells, Fenofibrate inhibits the activation of the mTOR pathway independently of the PI3K/AKT, MAPK, and AMPK pathways, but the mechanism underlying this effect remains unclear (Lian et al., 2017). Carver et al. (2011) reported that the use of the dual PI3K/mTOR inhibitor BEZ235 (also known as Dactolisib) and of enzultamide induces cell death in a *Pten*-deficient PCa mouse model, which results in ∼80% of tumor regression (Carver et al., 2011).

Stimulation of PCa cells with dihydrotestosterone has been described to induce eIF2α phosphorylation at Ser-51 in an AR-dependent way, thus shutting down global protein synthesis (Overcash et al., 2013). eIF4F assembly and activity has also been targeted by different drugs in prostate tumors or cells. In PC3 cultured cells, Cencic et al. (2009) showed that silvestrol impaired ribosome recruitment by affecting eIF4A activity and the composition of eIF4F complex. Silvestrol exhibits strong anticancer effects, such as increased apoptosis, decreased proliferation, and inhibition of angiogenesis in PCa xenograft animal models. It mediates its effects by preferentially inhibiting translation of malignancy-related mRNAs (Cencic et al., 2009). Jaiswal et al. (2018) have shown that treatment of CRPC C4-2B cells with the eIF4G/eIF4E complex formation inhibitor 4EGI-1 impairs prostate tumor progression. They also showed that treatment with 4EGI-1 sensitized CRPC cells to enzalutamide and bicalutamide, two antiandrogen chemotherapy agents currently used to treat PCa (Jaiswal et al., 2018). By using a mouse model of PCa, Hsieh et al. (2015) found that diminishing 4E-BP1 expression decreased resistance to the PI3K pathway inhibitor BKM120 in CaP cells (Hsieh et al., 2015). Additionally, PCa patients treated with BKM120 displayed increased 4E-BP1 abundance, indicating that 4E-BP1 may be associated with PCa progression and drug resistance (Hsieh et al., 2015).

## Outlook

Understanding the molecular processes underlying PCa will provide novel tools for both, its timely detection and the development of improved therapeutic strategies. To date, the United States Food and Drug Administration (FDA) has approved more than 20 pharmacological compounds for PCa treatment, most of which are hormonal modulators targeting the AR pathway. However, in most patients, advanced PCa develops resistance to androgen-deprivation therapies (Khemlina et al., 2015; Nevedomskaya et al., 2018). Due to the prolific studies in the field of dysregulation of translation in PCa, new molecules that can be chemically targeted are being rapidly identified. Most drugs tested in PCa models so far act on the signaling cascades controlling translation. Interestingly, other molecules targeting eIFs have shown activity in a myriad of different cancers but have not been yet tested in PCa. These include 4Ei-I, antisense eIF4E oligos, Hippuristanol, Pateamine A, 4E1R-Cat, 4E2R-Cat, and Rivavirin among others (Parsyan et al., 2012; Bhat et al., 2015; Malka-Mahieu et al., 2017; Siddiqui and Sonenberg, 2015). The next step should be to test these compounds in PCa.

Currently, although measurements of PSA in blood are the routine test for detection of possible PCa, the predictive value of PSA is at debate. In some studies, PSA has demonstrated a positive effect in the detection of potentially fatal cancer, but its value as a population screening tool can lead to poor diagnosis and treatments (Herget et al., 2016). In another study, a follow-up of 10 years, Martin et al. (2018) found that the single PSA screening intervention detected more PCa cases but had no significant predictive power of PCa mortality (Martin et al., 2018). Thus, there is a need to find more reliable markers that can complement the PSA test.

Genomics and epigenomics studies have led to the discovery of novel putative PCa biomarkers (Goh et al., 2014; Ngollo et al., 2014; Peng et al., 2014). Among these, the most promising molecule is the PCa antigen 3 (PCA3), a long non-coding RNA found overexpressed in more than 90% of prostate tumors (Kok et al., 2002). Different from the PSA marker, PCA3 has been detected neither in normal prostate tissues nor in prostatic hyperplasias, and PCA3 can be detected in urine samples from PCa patients with high certainty (Auprich et al., 2012). Another promising marker is the *TMPRSS2-ERG* fusion (Tomlins et al., 2005; Rubin et al., 2011), which is specific for PCa and can even be detected in precursor lesions such as prostate intraepithelial neoplasia (Mehra et al., 2008; Han et al., 2009). In the near future, this knowledge will be translated to pre-clinical and clinical phases, with multidisciplinary approaches for rigorous validation and future applications in PCa patients. As we have discussed here, the predictive value of hyperphosphorylated factors eIF4G, eIF4B, and eIF2α in PCa should also be validated soon. The next generation of markers should aim to efficiently detect PCa-specific circulating DNAs or microRNAs in fluids such as saliva or urine. They should also aim to detect early stages of this malady.

## Author Contributions

GH conceived, gathered information for the manuscript, and wrote most of the manuscript. JR, AP-T, LH, and MJ-R contributed to the writing as well as gathered information for the manuscript. AP-T and JR assembled Table 1 and Figure 1.

## Conflict of Interest Statement

The authors declare that the research was conducted in the absence of any commercial or financial relationships that could be construed as a potential conflict of interest.
